# The Pulse
*312. Clinical Manual Series. Published by Cassell and Co.


**Published:** 1890-04-26

**Authors:** 


### THE PRACTITIONER'S BOOKSHELF.

(Books for Review should be sent to The Editor, The Lodge, Porchester Square, W.)

### THE PULSE.*—By Dr. W. H. Broadbent.

Second Notice.

What we said about Dicrotism, in reviewing the earlier part of this work, applies also to the Pulsus Bisferiens, another, but very different, form of double beat. It is a condition by no means infrequent in advanced life, and we venture to think that a few more pages might have been well spent in its elucidation. It is a doubling of the beat which is scarcely perceptible to the finger, though brought out clearly enough in a good sphygmogram, occurring in aortic stenosis and atheromatous or other senile changes in the great vessels. It is just in cases such as this that the advantage derived from the sphygmograph is apparent.

We are glad to observe that Dr. Broadbent estimates the sphygmograph at its true worth, and, while admitting its great value, points out the difficulties and fallacies attending its use. Skill and practice are required in applying it, and, though in the hands of Marey, Sibson, and others it has done an immense deal for medical science, yet, on the other hand, "every important variety of the pulse revealed by the sphygmograph was recognised, described, and named before the Christian era." And surely resident physicians and clinical clerks can learn to do the same without its aid in the present day. To this end half a chapter is well spent on the method of detecting the qualities of the pulse by the fingers.

Successive chapters treat of the causes which modify the rate and tension of the pulse. The practical tone of the work can be seen from the manner in which these subjects are treated. Instead of dwelling on the physiological theories of the causes which influence the pneumogastric and sympathetic nerves, as a "laboratory-trained" physician would be apt to do, we find as causes which modify the rate of the pulse such realities as sleep, food, posture, excitement, peripheral obstruction, drugs, and a long list of diseased conditions, all of which modify the rate of the heart, and consequently of the pulse. Irregular pulses and the pulsus bigemminus also receive complete notice, and two interesting cases of "coupled heart-beats" are mentioned of patients in whom only every second beat of the heart reached the wrist with sufficient pressure to be perceived. In one of these, a gentleman aged 53, whose heart beat 72 whilst his pulse was only 36, Cheyne-Stokes respiration, a phenomenon of somewhat the same order occurred later on. The prognostic value of the various modifications, a point too often omitted from text books and manuals, is one which is dealt with in a thorough and masterly fashion. For instance, a rapid pulse in the early stage of acute specific fevers signifies a severe attack. An irregular pulse is frequently produced by excesses in tobacco, tea, and coffee, those luxuries, or shall we say necessaries, of our modern civilisation, and then it is an indication for their abandonment. But we are a little surprised to find that the author would sanction the administration of chloroform in cases of intermittent pulse, although the pulse be watched.

Interesting paragraphs are devoted to that obscure condition, cardiac palpitation, and persistent rapidity of pulse, for both of which digilatis still holds its own in treatment. Here and elsewhere there are valuable hints on treatment. An interesting case of the latter condition did remarkably well under treatment by strophanthus. The progress made by this case is well depicted by a series of sphygmographic tracings. There are 59 such tracings in the book, and they are well executed.

The concluding chapters treat of the pulse in aneurism, albuminuria, and affections of the nervous system. But enough has been said to indicate the scope and character of the work. Altogether, the book is one of the most practical, scholarly and masterly contributions to the pathology and treatment of the diseases of the circulatory system which has been produced of late years. We have heard this book spoken of as tough reading, but that is not the impression we have formed. There is no "padding," it is true, to dilute the valuable material it contains, but every point is clearly put and methodically arranged. Every word is worthy of perusal and reperusal. Such a work is highly welcome in these days of copious writing. There is but one regret we have, that there is not a more complete index. Improvement in this direction would greatly enhance its value as a work for reference.

* 312. Clinical Manual Series. Published by Cassell and Co.

